# Stepping Stones and Creating Futures intervention: shortened interrupted time series evaluation of a behavioural and structural health promotion and violence prevention intervention for young people in informal settlements in Durban, South Africa

**DOI:** 10.1186/1471-2458-14-1325

**Published:** 2014-12-29

**Authors:** Rachel Jewkes, Andrew Gibbs, Nwabisa Jama-Shai, Samantha Willan, Alison Misselhorn, Mildred Mushinga, Laura Washington, Nompumelelo Mbatha, Yandisa Skiweyiya

**Affiliations:** Gender & Health Research Unit, Medical Research Council, Private Bag X385, Pretoria, 0001 South Africa; School of Public Health, University of the Witwatersrand, Johannesburg, South Africa; HEARD, University of Kwa-Zulu Natal, Durban, South Africa; University of Pretoria, Pretoria, South Africa; Project Empower, Durban, South Africa

## Abstract

**Background:**

Gender-based violence and HIV are highly prevalent in the harsh environment of informal settlements and reducing violence here is very challenging. The group intervention Stepping Stones has been shown to reduce men’s perpetration of violence in more rural areas, but violence experienced by women in the study was not affected. Economic empowerment interventions with gender training can protect older women from violence, but microloan interventions have proved challenging with young women. We investigated whether combining a broad economic empowerment intervention and Stepping Stones could impact on violence among young men and women. The intervention, Creating Futures, was developed as a new generation of economic empowerment intervention, which enabled livelihood strengthening though helping participants find work or set up a business, and did not give cash or make loans.

**Methods:**

We piloted Stepping Stones with Creating Futures in two informal settlements of Durban with 232 out of school youth, mostly aged 18–30 and evaluated with a shortened interrupted time series of two baseline surveys and at 28 and 58 weeks post-baseline. 94/110 men and 111/122 women completed the last assessment, 85.5% and 90.2% respectively of those enrolled. To determine trend, we built random effects regression models with each individual as the cluster for each variable, and measured the slope of the line across the time points.

**Results:**

Men’s mean earnings in the past month increased by 247% from R411 (~$40) to R1015 (~$102, and women’s by 278% R 174 (~$17) to R 484 (about $48) (trend test, p < 0.0001). There was a significant reduction in women’s experience of the combined measure of physical and/or sexual IPV in the prior three months from 30.3% to 18.9% (p = 0.037). This was not seen for men. However both men and women scored significantly better on gender attitudes and men significantly reduced their controlling practices in their relationship. The prevalence of moderate or severe depression symptomatology among men and suicidal thoughts decreased significantly (p < 0.0001 and p = 0.01).

**Conclusions:**

These findings are very positive for an exploratory study and indicate that the Creating Futures/Stepping Stones intervention has potential for impact in these difficult areas with young men and women. Further evaluation is needed.

## Background

Informal settlements have a particularly high prevalence of major health problems, including HIV and sexually transmitted infections (STDs) and gender-based violence (GBV), affecting young people [1–6]. In sub-Saharan Africa they are an increasingly important settlement type, with over 70% of people in cities residing in informal settlements [7], including 4.4 million in South Africa [8]. Interventions to prevent HIV and gender-based violence in informal settlements are needed, but few interventions have been designed for this setting.

Interventions have been tested in South Africa to prevent gender-based violence and HIV. Stepping Stones, a participatory intervention for HIV prevention and strengthening relationship skills (see below), was evaluated with young men and women over a two year period in the rural Eastern Cape province in a randomised controlled trial (RCT) [9]. In this trial impact on HIV risk factors, the incidence of genital herpes (HSV-2) was significantly lower for men and women in the Stepping Stones arm than the control arm [9]. However, it reduced male self-reported perpetration of violence by 38% but did not reduce the experience of violence by women. It has been suggested that the limited impact Stepping Stones had on women (compared to men) may have resulted from their lack of external sources of power (such as economic leverage) [10].

Livelihood insecurity is a critical factor shaping HIV risk and vulnerability [11–13]. Poor women are more likely to engage in transactional sex [12, 14], have diminished agency in sexual relationships [15], are less able to leave abusive relationships and all of this reduces their capacity to protect themselves from intimate partner violence and HIV. Low educational attainment as well as low self-esteem and self-efficacy result in diminished capacity gain work or engage in strategies to craft stronger livelihoods [11]. To tackle these intersections, structural interventions, linking economic strengthening to gender-transformation are increasingly recognised as important [16]. The IMAGE study in rural South Africa combined a microfinance intervention with a gender-transformative intervention for women. After two years women in the intervention reported a 55% reduction in IPV experienced [17]. Similarly, a Village Savings and Loans Association (VSLA) intervention in the Ivory Coast for women added a couples intervention to reduce violence, consisting of 8 session ‘gender dialogue groups’ for women and their spouse, and evaluated this in a RCT (n = 1198 women randomised). While not showing positive results overall (overall the effect was non-significant and the reduction in physical and/or sexual IPV was 8%), it did show women who attended more than 75% of sessions with their male partner, experienced a 55% reduction in physical IPV, although this was not seen in the high adherence group for sexual IPV or the combined measure [18].

Yet similar interventions for young people have struggled to attain good outcomes; microfinance interventions tend not to work for young people as they have high levels of mobility [19], other approaches have sought to increase savings [20]. However one study with younger women in rural Uganda reported a reduction in coerced sex amongst female participants using a combination of economic strengthening interventions, including livelihoods training and microfinance [21]. There are also limitations to the reach of microfinance based interventions as these can only be built on existing well managed microfinance schemes, and these have limited coverage. Cash transfers have also been explored, but the major limitation of these is their cost. South Africa already has taken to scale social grants programmes, particularly child support grants up to age 18, without clear impact on HIV incidence and violence.

To see whether we could improve on the impact of Stepping Stones for women, and improve outcomes for both young men in very harsh circumstances, we developed a structural intervention, called Creating Futures. It aims to strengthen the livelihoods of young women and men in informal settlements without using microfinance or cash transfers. We implemented it in conjunction with the South African version of Stepping Stones, and conducted research with the goal of determining whether the combination of Creating Futures and Stepping Stones is a promising intervention to reduce gender-based violence and HIV risk among young men and women in two urban informal settlements in eThekwini municipality (Durban) of South Africa and worthy of impact evaluation.

## Methods

### Setting

This study was conducted in two urban informal settlements, Little Japan and Mbazwana, located in eThekwini District, KwaZulu-Natal, South Africa. Urban informal settlements are sites characterized by overcrowding, lack of decent housing, electricity, water and sanitation, and poor or no health care facilities and roads [3, 22]. Little Japan had a mix of government provided single room houses, alongside shacks and single room dwellings; it was located alongside a main highway and near a large township. Public transport by road (taxis) to the centre of Durban in took 25 minutes. Roads were untarred, formal electricity lacking and there were no inside toilets. Mbazwana, located on a steep hillside was relatively new and significantly poorer than Little Japan. Central Durban was two taxis and at least 45 minutes away, and electricity, pathways and toilets were lacking.

### Participants

We recruited 232 out-of-school young people (aged 18 to 34, with most under 30), of these, 110 were men and 122 were women. Recruitment was done by a Durban based non-governmental organization (NGO), Project Empower, which specialised in delivering a range of intervention related to economic empowerment in informal settlements. Given the challenges of accessing urban informal settlements, including challenging political and social environments, Project Empower used their experience to access and continue to secure ongoing access to urban informal settlements. They recruited participants because they could negotiate complex political arrangements in each setting, where attempts to ‘mobilise’ many young people were often cast as a political process. Project Empower also co-trained, managed and supervised the facilitators throughout the intervention.

Upon accessing the communities, Project Empower handed out flyers with information about the study and a contact number for those interested in participating to call or send text messages. Those who made telephonic contact were invited for a face-to-face meeting where they were provided more information about the study. Snowball sampling technique was also used to recruit other participants.

### Intervention and implementation

The livelihoods intervention (Creating Futures (available for web download) [23]) that was combined with Stepping Stones for evaluation was developed by members of the study team [24]. Creating Futures is a facilitated group intervention covering eleven, three-hour sessions in single-sex groups of approximately twenty people. It was developed by drawing on sustainable livelihoods theory and practice [25, 26]. This work finds that people build and maintain their means of making a living and surviving by drawing on a range of resources which have been distinguished broadly into five capitals: financial capital, natural capital (emanating from the natural environment), human capital (such as education, health, work experience), physical capital (such as built environment assets), and social capital (emanating from our interactions between and within individuals and groups) [27–29]. These capitals not only offer the raw material for fashioning livelihoods, but can also encompass elements that constrain livelihood choices and explain many of the inequities between individuals as well as communities [26]. The ability to draw on – as well as build- a combination of resources to make a living is fundamental to finding pathways out of poverty and vulnerability that might decrease exposure to HIV related risk [19].

In Creating Futures, participants engage in participatory learning activities to reflect on and critically analyse their livelihoods and develop skills for strengthening them, using existing resources in their environment. The key sessions cover: my life and the resources I have used in my life and livelihood, setting medium term livelihood goals and the need for assets for livelihoods and coping with crises; social resources for livelihoods (trust and community participation); education and learning including past experiences and how to build on these; getting and keeping jobs including work expectations and how ones on own behaviours may impede or increase our ability to get a job and to keep it, appropriate work opportunities and increase owns own ability to market ones skills and apply for work and overcome challenges in job seeking and maintaining a job; income generating activities and how to identify viable business opportunities, the resources necessary to respond to such opportunities, basic business principles and business risks; saving and coping with shocks including spending patterns and strategies for saving, and causes and consequences of getting into debt and ways of overcoming debt.

The third edition of the South African adaptation [30] of Stepping Stones [31] uses participatory learning approaches, including critical reflection, role play, and drama and draws the everyday reality of participants’ lives into the sessions. It is an HIV and violence prevention programme that aims to build more gender-equitable relationships with better communication between partners. It is delivered to single sex groups, which are run in parallel but come together for peer group meetings to facilitate communication and understanding. It has 10 three hour sessions and can be used with all ages [30]. These cover gender and peer influences our actions; sex and love; conception and contraception; STIs and HIV; safer sex and condoms; GBV; motivations for behaviour (including influences of alcohol and poverty); and communication skills.

The implementation of the combined intervention was undertaken by an NGO Project Empower. They employed facilitators who had completed secondary school, and some had experience in the health sector and in facilitation, but also trained them on gender attitudes, norms and inequalities, HIV and AIDS, sexual and reproductive health, and facilitation skills. Creating Futures was designed to link directly after and build on the preparatory work of Stepping Stones. As such the interventions were run in sequence – that is all 10 sessions of Stepping Stones were completed, before 11 sessions of Creating Futures were conducted. Sessions were all approximately three hours long, delivered twice a week, over approximately 12 weeks. They were delivered by trained facilitators, who were similar in age to the participants, but were slightly better off materially. They had all completed high-school education and a few of them had prior experience of facilitating interventions.

Sessions were undertaken in a central location in Durban, near a taxi rank. Participants received no reimbursement for attending, however the project covered transport costs incurred by participants and provided basic refreshments during sessions. Given the challenging context, attendance was mixed, with many continuing to seek work, returned to ‘rural’ homes for short periods and some struggled to access the taxi fare [32]. Drawing on incomplete facilitator records, we estimated an attendance rate of 60%, among those who attended any sessions.

### Study design

We employed a shortened interrupted time-series design with two data collection points at baseline which were two weeks apart. We had follow-up interviews 28 weeks and 58 weeks post-baseline. This was a proof of concept study, not an impact evaluation, and so the choice of study design and sample size were driven by concerns related to affordability. This quantitative approach complimented qualitative research, which used three focus groups and 19 interviews with men (and in some cases their partners) before and 6 and 12 months after the intervention [32–34].

Data were collected using self-completed questionnaires. The questionnaires for men and women were somewhat similar and had standard scales that had been validated and used in other studies in South Africa [31, 35]. We assessed the demographic and socio-economic background of the participants, sexual behaviour, gender attitudes and participation in crime. Gender attitudes were measures on a 19 item scale (with a 4 point Likert response from Strongly Agree to Strongly Disagree) (Cronbach’s alpha 0.786). The measures drew on the Gender Equitable Men scale (GEM) and items developed and tested in South Africa and the whole scale reflected questions used in the UN Multi-country Study of Men and Violence in Asia and the Pacific [36]. The work stress questions were taken from the Images study [37] and consisted for 4 items with a Likert scale (alpha 0.622). Men were asked about the circumstances under which they had had sex with any woman, relationship control practices [38], about ever perpetrating physical and sexual violence on any intimate partner and sexual violence on any woman [39]. Women were asked about the circumstances under which they had had sex with any man, experiences of controlling practices from their partner, and of physical and sexual violence by an intimate partner [40].

The mental health of participants was explored using CES-D scale to assess depressive symptomatology [41]. Engagement in transactional sex was explored for both men and women [35]. We adapted the AUDIT scale [42] and assessed participants’ alcohol and drug use.

Ethical approval was given by the research ethics committees of the South African Medical Research Council and the University of KwaZulu-Natal. The permission to recruit participants within the communities was granted by the community gatekeepers. Written informed consent was obtained from all participants. Privacy in the administration of the questionnaires was ensured, and confidentiality and anonymity maintained. At each data collection point, participants who completed a questionnaire were given R50 (~$5).

All questionnaires were entered into Epi Info 7 and the data was analysed using Stata version 12.0. Analysis was by intention to treat, thus we included in the follow-up all participants who were initially enrolled into the intervention irrespective of attendance. We analysed the data for male and female participants separately. Descriptive statistics were used to describe the proportion of participants who reported each characteristics or behaviour at each time point. For scales, scores were derived by summing the response. For all continuous variables, the mean is presented for each time point. The dataset collected at each of the time points were merged in long format. We then built a random effects regression model with each individual as the cluster, and measured the slope of the line across the time points to determine trend. This was done for each variable.

## Results

In total 110 men completed the baseline, 93 round 2, 105 round 3, and 94 completed the last assessment (85.5% of those enrolled). Among women, 122 completed the baseline, 113 round 2, 116 round 3, and 111 were interviewed at the 4th data point (90.2% of those enrolled). Loss to follow up was due to three participant deaths (unrelated to the study) and one male participant was in jail; others were untraceable, and given high levels of migration, we assume they moved out of the study community and had changed cell phone numbers.

Participants were mostly aged 18–30 years. One man was 17 and two women were over 30 (33 and 34). Nearly half of men (45.4%) and a quarter of women (23.6%) had completed high school (grade 12) (Table 1). Most participants had a long-term partner but were not married or cohabiting. Two-thirds of women and a third of men had a biological child(ren). Among those with children, a third had more than one and one in ten had more than two children (10% of men and 13.4% of women). At baseline, two-thirds of men and a third of women had worked or earned in the previous 12 months.

**Table 1:** **Characteristics of the participants enrolled for round one**

	Male	Female
	% (n = 110)	% (n = 122)
Age group: <20 yrs	20	31.2
20-24	66.4	48.4
25-29	13.6	18.9
>30	0	1.6
Highest school grade: <10	20	24.4
10	7.3	13
11	27.3	39
12	45.4	23.6
Post-school course	20	15.5
Mother has died	23.9	26
Father has died	49.5	55.3
Partnership status: married	0.9	0.8
Cohabiting	14.6	8.1
Has a girl or boy friend	71.8	72.4
No current partner	12.7	18.7
Ever had a child or fathered	36.4	66.7
# children: >1	57.5	59.8
2	32.5	26.8
>2	10	13.4
Worked or earned in last 12 m	65.2	36.1

The socio-economic indicators measured in the study are presented in Table 2. At baseline mean earnings in the past month of men were R 411 (~$40) and of women was R 174 (~$17). By the fourth round mean earnings of men had increased by 247% to R1015 (~$102) and of women by 278% to R 484 (about $48). The test for trend across the time points showed that this increase was highly significant (p < 0.0001). At baseline 10.9% of men and 11.4% of women were currently studying. These proportions were higher at round 4, with 17.9% and 15.3% of men and women studying, but the trend was not significant. At baseline women scored higher than men on a measure of their attempts to strengthen their livelihoods, but their score did not change over the year, however for men it increased significantly (p < 0.0001). The work-related stress scale showed a similar pattern, with men’s stress reducing over the year (p = 0.039) but not women’s, although there may have been a reduction at 6 months, which was not sustained. However, a measure of feelings about work situation showed significant improvement (p < 0.0001) for both women and men.

**Table 2:** **Socio**-**economic indicators**

	Pre-intervention				Post-intervention					
	Baseline		Round 2		Round 3		Round 4		Male	Female
	Male (n = 110)	Female (n = 123)	Male (n = 93)	Female (n = 113)	Male (n = 105)	Female (n = 116)	Male (n = 94)	Female (n = 111)	pvalue*	pvalue
Mean earnings last month (Rands)	411	174	296	113	738	323	1015	484	<0.0001	<0.0001
Currently studying	10.9	11.4	13	8.8	11.4	12	17.9	15.3	0.133	0.127
Frequency of livelihood strenghtening efforts (score)	17.1	20.3	18	20.7	18.2	19.4	19.3	20.1	<0.0001	0.29
Work stress										
Work stress mean score (high = less stress)	7.43	8.01	7.76	8.05	7.64	8.46	8.18	7.88	0.039	0.94
Feelings about work situation mean score (high = feeling better)	9	9.8	10.3	9.6	10.36	10.63	11.04	10.75	<0.0001	<0.0001
Ability to support children										
Financially supporting kids	40	54.1	44.1	53.1	44.76	58.97	46.88	61.26	0.42	0.03
Receiving a grant	9.3	48.8	14.1	46.9	16.35	52.99	10.53	56.88	0.46	0.009
economic hardship & crime										
Hungry every day or week:	24.5	24.4	38.7	35.4	28.85	21.37	21.88	31.82	0.545	0.7
Borrowing food or money weekly or more often	17.4	27.6	18.5	17.7	15.24	18.97	12.37	24.32	0.26	0.5
Stole in last month due to lack of food or money	33.9	47.2	33.7	45.1	26.67	35.04	24.74	35.14	0.039	0.005
Crime participation score (high = more crime)	0.982	0.76	1.34	0.885	0.97	0.76	1.15	0.77	0.51	0.85
Very diffficult to find R 200 in an emergency	40.9	68.3	35.5	57.5	36.19	46.15	22.68	42.34	0.002	<0.0001
Social capital										
Any club or group involvement	48.2	22.8	45.7	22.1	36.19	26.5	52.1	31.2	0.77	0.07
Active in church	50	41.4	40.2	38.9	42.86	39.32	41.7	33.6	0.27	0.16
Community cohesion score (high = less social cohesion)	8.96	9.28	9.68	9.57	9.5	9.47	9.42	9.67	0.12	0.21

*P value is from a random effects regression model with each individual as the cluster, and measured the slope of the line across the time points to determine trend.

At baseline 40% of men and 54% of women with children said they financially supported them. This increased to 47% of men and 61% of women after the 12 months. An increase that was significant for women (p = 0.03). 9.3% of men and 48.8% of women were receiving a child or foster care grant for children in their care at baseline, and this increased significantly for women to 56.9% (p = 0.009) after one year.

A quarter of men and women indicated that they went without food for lack of money every week or day. This did not change over the year. There was also no change in the proportion who borrowed money or food from neighbours each week, or more often. In all 33.9% of men and 47.2% of women said at baseline that they had stolen in the previous month due to lack of food or money. This proportion substantially reduced over the 12 month period. However a 12 item scale measuring participation in a range of different forms of crime showed no overall change. Perceived ability to mobilise money (R200 or ~ US$20) in an emergency improved over one year. At baseline 40.9% of men and 68.3% of women indicated this would be very difficult, the proportion was very much lower at 12 months and this was significant (p < 0.002 and p < 0.0001) for men and women respectively.

The questionnaire included three measures of social capital. There was a suggestion that women may have become more involved in clubs or groups (from 22.8 to 31.9%) and less active in church (from 41.4 to 33.6%) over the year, but neither change was significant. There was no change among men on either of these measures. Neither men nor women perceived change in community cohesion.

The men and women’s gender attitudes and prevalence of experience of and perpetration of GBV are shown in Table 3. Measured on a gender attitudes scale, there was evidence over the 12 months that both men’s and women’s gender attitudes become more equitable (both significant). There was some improvement in a measure of relationship control over the 12 months, with this highly significant for men but not for women. Physical intimate partner violence (IPV) perpetration by men in the prior 3 months was less prevalent at round 4 than the preceding three rounds, but the trend was not significant. For women there was no clear change trend in experience of physical IPV. For men, there was no trend of change in the prevalence of perpetration of sexual IPV, but for women there was a significant reduction of sexual IPV in the past three months. There was no change in the prevalence of non-partner rape perpetration for men. There was a significant reduction in experience of the combined measure of physical and/or sexual IPV in the prior three months from 30.3% to 18.9%, a 38% reduction (p = 0.037) in women.

**Table 3:** **Gender indicators**

	Pre-intervention				Post-intervention					
	Baseline		Round 2		Round 3		Round 4		Male	Female
	Male	Female	Male	Female	Male	Female	Male	Female	pvalue*	pvalue
Gender attitudes scale mean score (high = more equitable)	50.8	53.7	50.6	53.3	51.23	54.03	52.89	55.29	0.007	0.01
Relationship control scale (high = more equitable)	19.4	22.2	20.3	21.9	21.21	22.38	21.74	22.82	<0.0001	0.11
Physical IPV in last 3 m**	16.5	27.9	16.5	18.3	17.3	25.6	12.5	18.0	0.49	0.12
Sexual IPV in last 3 m	14.7	9.8	16.5	12.5	12.5	7.7	13.5	3.6	0.69	0.033
Rape of a non-partner in last 3 m (men only)	2.8		6.7		4.8		6.3		0.29	
Physical or sexual IPV in last 3 m	23.9	30.3	25.3	25.7	26.0	27.4	21.9	18.9	0.86	0.037

*P value is from a random effects regression model with each individual as the cluster, and measured the slope of the line across the time points to determine trend.

**all IPV measures are of perpetration by men and experience by women.

A series of health measures were examined (Table 4). The prevalence of moderate or severe depression symptomatology decreased substantially in men (from 74.8% to 53.4% p < 0.0001). This was not seen in women. There were significant improvements in both men and women, however, in a scale assessing satisfaction with life circumstances. At baseline 25.5% of men and 22.3% of women had had suicidal thoughts in the previous month, and this reduced to 9.5% and 12.7% respectively at one year. The change was significant for men but not for women.

**Table 4:** **Health indicators**

	Pre-intervention				Post-intervention					
	Baseline		Round 2		Round 3		Round 4		Male	Female
	Male	Female	Male	Female	Male	Female	Male	Female	pvalue*	pvalue
Depression: moderate/severe symptomatology	74.8	72.0	64.1	67.0	57.1	77.1	53.4	70.9	<0.0001	0.79
Life circumstances score (low = better)	13.3	14.1	12.6	13.3	12.68	13.08	11.65	13.05	<0.0001	0.002
Suicidal thoughts in last 4 wks	25.5	22.3	18.3	16.8	17.1	21.4	9.5	12.7	0.001	0.1
Alcohol problem in last 12 m	42.9	26.6	51.8	29.0	48.2	32.3	49.1	35.5	0.36	0.049
Quarrel because of drink in last 3 m (among drinkers)	31.2	40.9	27.4	29.6	25.7	30.8	27.5	22.6	0.56	0.026
Drug use in last 3 m	33.6	17.2	33.3	10.6	29.5	8.6	30.9	18.2	0.5	0.88
Had a HIV test	57.3	81.8	54.8	86.7	56.2	87.2	69.1	81.1	0.044	0.99
Last sex with main partner	50.0	80.3	51.6	87.0	62.5	82.9	61.7	86.9	0.027	0.32
Condom on last sex	69.4	55.6	72.5	54.6	61.5	59.5	71.4	61.7	0.8	0.18
Transactional sex in last month	15.9	10.3	14.6	13.8	15.4	18.6	16.0	13.1	0.85	0.25

*P value is from a random effects regression model with each individual as the cluster, and measured the slope of the line across the time points to determine trend.

A measure of problem alcohol drinking in the past 12 months did not change for men. It did change significantly for women, in the direction of an increased proportion (26.6% at baseline to 35.5% at round 4). However, among women who drank alcohol, the proportion quarrelling with their partners over their drinking declined significantly from 40.9% to 22.6% (p = 0.026). There was no change for men. The proportion of men and women who used drugs in the past three months did not change.

At baseline 57.3% of men had had an HIV test and by round 4 this was 69.1%, a significant change. The prevalence was higher for women (81.8% at baseline) and did not change. About 50% of men and 80.3% of women had last had sex with their main partner at baseline. The proportion increased significantly for men by round 4 to 61.7%. There was no change for women. The proportion of men who had used a condom at last sex did not change, but there was an underlying trend of increase for women (from 55.6 to 61.7%) but this was not statistically significant. There was no change across the study in the proportion of men and women who had had transactional sex in the past month.

## Discussion

We presented here a pilot study of the Stepping Stones and Creating Futures combined intervention, with the outcome assessed using a shortened interrupted time series design. Overall the results suggest the intervention had an impact on livelihoods, specifically women and men improved their monthly earnings, felt less stressed about their work situation, stole less because of lack of money, and were more able to access money in an emergency. Furthermore, men increased their livelihood strengthening efforts and women increased their access to child support grants and supported their children more. There were also a range of positive changes in gender-related and violence measures. Both women and men had more gender-equitable attitudes and men reduced controlling behaviours towards partners, while women felt less controlled by partners. In addition, women experienced less sexual IPV and sexual and/or physical IPV. Reading of the qualitative analysis complemented these observed statistical changes. These are summarised in the following extract from the abstract of the main qualitative paper:

*Our data suggests that rather than a wholesale reconstruction of masculinity a more subtle shift was seen with men moving away from more* '*harmful*' *aspects of a dominant youth masculinity towards a form of masculinity in which male power is buttressed by economic provision and attempting to setup stable* '*households*', *drawing on aspects of a* '*traditional*' *masculinity. Working with men on their livelihoods certainly at an instrumental level*, *did encourage participation in the intervention. Beyond encouragement*, *men*'*s improving livelihoods appeared to afford men the opportunity to materially demonstrate the social changes* - *shifts in masculinity* - *they were seeking to enact* [33].

More widely men’s and women’s broader health showed improvements. Women’s and men’s perceived life circumstances improved. As a group, men reduced symptoms of depression and suicidal thoughts and had more HIV tests. In addition, a greater proportion of men reported the person they had sex with was their main partner. Women reduced quarrelling over their drinking, but more appeared to have drank heavily. Thus the intervention appeared to have strengthened livelihoods, had positive impact on gender relations and improved many aspects of mental health. These findings are broadly supported by the qualitative research [33].

Creating Futures draws on a sustainable livelihoods framework, which identifies five capitals: financial, human, social, physical and natural that people draw on to make a livelihood. The intervention seeks to bolster these capitals and thus strengthen participants’ livelihoods. Our findings suggest evidence of success in building financial capital, with higher monthly incomes and more women accessing child support grants. The impact of this was tangibly measured in the greater proportion of women supporting their children, and fewer men and women stealing for lack of money or food. There may have been a positive trend in the direction of greater human capital as the proportion of men and women at round 4 studying was higher, if not statistically significant. Shock resilience, as measured by perceived ability to access R200 for an emergency, improved for both men and women. It was not clear if the intervention increased social capital, it may have done so for women but the trend was not statistically significant (p = 0.07).

These findings are important as Creating Futures is a structural intervention that does not require large sums or capital, unlike cash transfers (not withstanding claims that these may be cost effective [43]) and microfinance. Microfinance has shown mixed success with adolescents and requires functioning microfinance projects [19, 21, 44]. It is interesting that the adolescent girls in Uganda showed a substantial reduction in their experience of unwanted sex after the intervention, mirroring the reduction of sexual IPV reported by women in our study. There is a need for further evaluation, but this study suggests that Creating Futures may represent a new generation of structural interventions which may be of value in South Africa’s informal settlements, as well as potentially in other countries, and have potential for scalability, within and beyond South Africa, because it does not require capital beyond the costs of delivery of the intervention.

Like the previous evaluation of Stepping Stones in South Africa [45], there was a positive impact on gender relations and violence. However the nature of this differed from that in the earlier evaluation. The larger Stepping Stones study showed no impact on experience of IPV among women [31], which contrasted with our prominent finding of statistically significant decreases in women’s experience of sexual and/or physical IPV and sexual IPV. This supports a growing body of evidence that suggests women require change in their material circumstances in order to be able to use knowledge from gender-transformative programmes to reduce violence; most clearly seen in the IMAGE study [17]. This study also showed men’s controlling practices reduced. This is important as these have been shown to increase women’s risk of HIV incident infections [46]. However we did not find a reduction in violence perpetration; the reason could be that the follow up was too short, as in the first Stepping Stones evaluation impact was seen at 24, not 12 months [45]. We did note round 4 prevalence was lower (12.5%) than the other three rounds (if not significantly so). This may have been the start of a trend, and it was notable that the other three measures were extremely close to each other (16.5%, 16.5% and 17.3%). It is possible that Stepping Stones impacted differently in the informal settlement context as the prevalence of physical and sexual violence perpetration are very high and there is considerable evidence that the harsh environment resulted in more emphasised masculinities that were more strongly predicated on control of women and where violence was a ready resort in conflict of all forms [34, 47, 48]. These social norms may be more difficult to change.

The intervention appeared to positively impact on mental health, reducing depression and suicidality for men, and improving perceived life circumstances. This was also indicated in the first Stepping Stones evaluation findings, where depression in men may have somewhat reduced (p = 0.1). These are important findings and help to support an overall picture of benefit from the intervention. In the first Stepping Stones study no increase in women’s drinking was seen. However the participants were in school, the setting was conservative and very few drank. The finding in our study suggests that drinking more is a consequence of having a higher income. It is obviously concerning, but the reduction in quarrelling over women’s drinking may suggest that women had more skills to avoid some of the common adverse consequences for them. Research from Cape Town with young men and women shows that the impact of drinking on IPV risk is mediated through poor conflict skills [49]. The increase in willingness to test for HIV was not reported in the first Stepping Stones report, but was suggested by accompanying qualitative research [10]. It is encouraging to see this confirmed here. The failure to impact on women’s HIV testing may be explained by the levels already being very high and much higher than those of men, likely due to the fact that HIV testing is increasingly common in antenatal settings in South Africa. In this study two-thirds of the women had biological children and it is therefore likely most would have tested during pregnancy.

Qualitative research findings among men in this intervention show that many reported having better, less conflictual relationships with their main partners and as a result spending more time with them and less with other sexual partners [33]. The report that a higher proportion of last sexual partners were main partners seems to confirm this finding and points to an effect of the intervention on sexual health, as well as gender relations.

The study had limitations. The sample size was small and so the power to detect change was limited. There was no control group and so we cannot be sure of underlying trends, although the relatively short period of the study (one year of follow up) makes it unlikely that underlying trends would have been of great change among participants. Measurement error and bias and reporting changes due to repeated observation are problems with intervention evaluations and normally control groups provide a comparator to the intervention arm in this regard. The use of a shortened interrupted time series, however, helped considerably in understanding the repeatability of the measures, with the two baselines enabling changes which are an artefact of repeated assessment to become visible. This is especially important when the underlying construct could have been subject to disclosure bias (e.g. in illegal activities of crime participations and rape perpetration). Both of these measures provided lower estimates at the first baseline than the second, suggesting there was some disclosure bias. There was some loss to follow up across time, which could have influenced the findings but we are unable to know in which direction.

## Conclusions

This study has demonstrated that the combined Stepping Stones and Creating Futures intervention has potential to strengthen livelihoods, improve gender relations, reduce violence and improve mental health among young people in South Africa’s informal settlements. This is a very vulnerable group given the very high prevalence of unemployment, HIV incident infections and violence in these areas. This study has shown that this intervention deserves further evaluation and may have the potential to substantially improve the lives of a very important section of South African society who live in particular harsh circumstances.

## References

[1] Thomas L, Vearey J, Mahlangu P (2011). Making a difference to health in slums: an HIV and African perspective. Lancet.

[2] Hunter M (2006). Informal settlements as spaces of health inequality: the changing economic and spatial roots of the AIDS pandemic, from apartheid to neoliberalism. Centre Civil Soc.

[3] Vearey J, Palmary I, Thomas L, Nunez L, Drimie S (2010). Urban health in Johannesbrug: the importance of place in understanding intra-urban inequalities in a context of migration and HIV. Health Place.

[4] UNFPA (2007). State of world population: unleashing the potential for urban growth.

[5] Shisana O, Rehle T, Simbayi LC, Parker W, Zuma K, Bhana A, Connolly C, Jooste S, Pillay V (Eds): *South African National HIV Prevalance, HIV incidence, Behaviour and Communication survey* Cape Town: HSRC press; 2005.

[6] Amunyunzu-Nyamongo M, Okeng'O L, Wagura A, Mwenzwa E (2007). Putting on a brave face: the experiences of women living with HIV and AIDS in informal settlements of Nairobi, Kenya. AIDS Care.

[7] WHO (2008). Our cities, our health, our future: acting on social determinants for health equity in urban settings.

[8] HDA (2011). Informal Settlements - KwaZulu-Natal.

[9] Jewkes R, Nduna M, Levin J, Jama N, Dunkle K, Khuzwayo N, Koss M, Puren A, Wood K, Duvurry N (2006). A cluster randomised controlled trial to determine the effectiveness of Stepping Stones in preventing HIV infections and promoting safer sexual behaviour amongst youth in rural Eastern Cape, South Africa: trials design, methods and baseline findings. Trop Med Int Health.

[10] Jewkes R, Wood K, Duvurry N (2010). “I woke up after I joined Stepping Stones”: meanings of an HIV behavioural intervention in rural South African young people’s lives. Health Educ Res.

[11] Hunter M (2010). Love in the time of AIDS: inequality, gender, and rights in South Africa.

[12] Weiser SD, Leiter K, Bangsberg DR, Butler LM, Korte FPD, Hlanze Z, Phaladze N, Iacopino V, Heisler M (2007). Food insufficiency is associated with high-risk sexual behavior among women in Botswana and Swaziland. Plos Medicine.

[13] Kamndaya M, Thomas L, Vearey J, Sartorius B, Kazembe L (2014). Material deprivation affects high sexual risk behavior among young people in urban slums.

[14] Dunkle KL, Jewkes RK, Brown HC, Gray GE, McIntyre JA, Harlow SD (2004). Transactional sex among women in Soweto, South Africa: prevalence, risk factors and association with HIV infection. Soc Sci Med.

[15] Jewkes R, Morrell R (2010). Gender and sexuality: emerging perspectives from the heterosexual epidemic in South Africa and implications for HIV risk and prevention. J Int AIDS Soc.

[16] Gupta GR, Parkhurst JO, Ogden JA, Aggleton P, Mahal A (2008). Structural approaches to HIV prevention. Lancet.

[17] Pronyk P, Hargreaves JR, Kim JC, Morison LA, Phetla G, Watts C, Busza J, Porter JD (2006). Effect of a structural intervention for the prevention of intimate partner violence and HIV in rural South Africa: a cluster randomised trial. Lancet.

[18] Gupta J, Falb KL, Lehmann H, Kpebo D, Xuan Z, Hossain M, Zimmerman C, Watts C, Annan J (2013). Gender norms and economic empowerment intervention to reduce intimate partner violence against women in rural Cote d'Ivoire: a randomized controlled pilot study. BMC Int Health Hum Rights.

[19] Gibbs A, Willan S, Misselhorn A, Mangoma J (2012). Combined structural interventions for gender equality and livelihood security: a critical review of the evidence from southern and eastern Africa and the implications for young people. J Int AIDS Soc.

[20] Austrian A, Muthengi M (2013). Safe and smart savings Products for vulnerable adolescent girls in Kenya and Uganda.

[21] Bandiera O, Buehren N, Burgess R, Goldstein M, Gulesci S, Rasul I, Sulaiman M (2012). Empowering adolescent girls: evidence from a randomized control trial in Uganda.

[22] Vearey J (2011). Challenging urban health: towards an improved local government response to migration, informal settlements, and HIV in Johannesburg, South Africa. Global Health Action.

[23] Misselhorn A, Jama Shai N, Mushinga M, Washington L, Mbatha N (2012). Creating futures.

[24] Misselhorn A, Jama-Shai N, Mushinga M, Washington L (2014). Creating futures: supporting young people in building their livelihoods. Sex Educ.

[25] Scoones I (2009). Livelihoods perspectives and rural development. J Peasant Stud.

[26] Mosoetsa S (2011). Eating from one pot: the dynamics of survival in poor South African households.

[27] Bebbington A (1999). Capitals and capabilities: a framework for analyzing peasant viability. Rural Livelihoods and Poverty. World Dev.

[28] Chambers R, Conway GR (1991). Sustainable rural livelihoods: practical concepts for the 21st century.

[29] Scoones I (1998). Sustainable rural livelihoods: a framework for analysis. In: IDS working paper 72.

[30] Jewkes R, Abrahams N (2002). The epidemiology of rape and sexual coercion in South Africa: an overview. Soc Sci Med.

[31] Welbourn A (1995). Stepping Stones.

[32] Gibbs A, Jewkes R, Mbatha N, Washington L, Willan S (2014). Jobs, food, taxis and journals: complexities of implementing Stepping Stones and Creating Futures in urban informal settlements in South Africa. AJAR.

[33] Gibbs A, Jewkes R, Sikweyiya Y, Willan S (2014). Reconstructing Masculinity? A qualitative evaluation of the Stepping Stones and Creating Futures intervention in urban informal settlements in South Africa.

[34] Gibbs A, Sikweyiya Y, Jewkes R (2014). 'Men value their dignity': securing respect and identity construction in urban informal settlements in South Africa. Glob Health Action.

[35] Dunkle K, Jewkes R, Brown HC, Yoshihama M, Gray GE, McIntyre JA, Harlow SD (2004). Prevalence and patterns of gender-based violence and revictimization among women attending antenatal clinics in Soweto, South Africa. Am J Epidemiol.

[36] Fulu E, Warner X, Miedema S, Jewkes R, Roselli T, Lang J (2013). Why do some men use violence against women and how can we prevent it.

[37] Barker G, Contreras M, Heilman B, Singh A, Verma R, Nascimento RRR (2011). Evolving men: initial results from the international men and gender equality survey (IMAGES).

[38] Pulerwitz J, Gortmaker S, Dejong W (2000). Measuring sexual relationship power in HIV/STD research. Sex Roles.

[39] Jewkes R, Sikweyiya Y, Morrell R, Dunkle K (2009). Understanding men's health and use of violence: interface of rape and HIV in South Africa.

[40] Garcia-Moreno C, Jansen HAFM, Ellsberg M, Watts C (2005). WHO multi-country study on women's health and domestic violence against women: initial results on prevalence, health outcomes and women's responses.

[41] Radloff L (1977). The CES-D scale: a self report depression scale for research in the general population. Appl Psychol Meas.

[42] Saunders JB, Aaslando O, Babor T, De La Fuente JR, Grant M (1993). Development of the Alcohol Use Disorders Identification Test (AUDIT): WHO collaborative project on early detection of persons with harmful alcohol consumption. Addiction.

[43] Handa S, Halpern CT, Pettifor A, Thirumurthy H (2014). The government of Kenya’s cash transfer program reduces the risk of sexual debut among young people age 15–25. PLoS ONE.

[44] Dunbar MS, Maternowska MC, Kang MS, Laver SM, Mudekunye-Mahaka I, Padian NS (2010). Findings from SHAZ!: a feasibility study of a microcredict and life-skills HIV prevention intervention to reduce risk among adolescent female orphans in Zimbabwe. J Prev Int Commun.

[45] Jewkes R, Nduna M, Levin J, Jama N, Dunkle K, Puren A, Duvvury N (2008). Impact of Stepping Stones on incidence of HIV, HSV-2 and sexual behaviour in rural South Africa: cluster randomised controlled trial. Br Med J.

[46] Jewkes R, Dunkle K, Nduna M, Shai N (2010). Intimate partner violence, relationship gender power inequity, and incidence of HIV infection in young women in South Africa: a cohort study. Lancet.

[47] Bourgois P (1996). In search of masculinity - Violence, respect and sexuality among Puerto Rican crack dealers in East Harlem. Br J Criminol.

[48] Mathews S, Jewkes R, Abrahams N (2011). ‘I had a hard life”: exploring childhood adversity in the shaping of masculinities among men who killed an intimate partner in South Africa. Br J Criminol.

[49] Russell M, Cupp P, Jewkes R, Gevers A, Mathews C, LeFleur-Bellerose C, Small J (2014). Intimate partner violence among adolescents in Cape Town, South Africa. Prev Sci.

[CR50] The pre-publication history for this paper can be accessed here:http://www.biomedcentral.com/1471-2458/14/1325/prepub

